# The Hospital Nursing Supplement

**Published:** 1894-06-30

**Authors:** 


"The Hospital" Nursing Mirror.

Being the Extra Nursing Supplement of "The Hospital" Newspaper.

[Contributions for this Supplement should be addressed to the Editor, The Hospital, 428, Strand, London, W.C., and should have the word "Nursing" plainly written in left-hand top corner of the envelope.]

### News from the Nursing World.

### GOOD TIDINGS!

The birth of an infant Prince at White Lodge, Richmond, on 23rd inst., has been a cause of much joy to the Queen (his great grandmother), as well as to the nation. Her Majesty visited H.R.H. the Duchess of York on Tuesday, and herself nursed the healthy little baby, who is the direct heir to the English Throne. The Prince of Wales paid an early visit of congratulation to the Duke of York—"our Princess" was with the Duchess of York throughout—and the bulletins issued daily are as favourable as even the most loyal of nurses could desire. Members of the Pension Fund will join with one of our correspondents in the desire to offer humble but heartfelt congratulations to their president H.R.H. the Princess of Wales on so joyous an occasion.

### ERRORS OF DIET.

"Doctor, my girl's sick to death of milk an' beef tea and fish, can't I give her something different?" asked a patient's mother one morning when the doctor called. "Well, you must be very careful," said he decidedly. "You know your daughter has had a very severe illness, and will need careful feeding for a long time yet. What do you want to give her?" he added, doubtfully. "Well, sir, I'll be guided by you; but the squire's wife was in yesterday—she's wonderful fond of Kate—and she said she'd take it as a kindness if I'd let her send anything as the doctor ordered for the girl. So please to give it a name, sir." The doctor looked thoughtful. "I don't think a sweetbread would hurt her if it was well cooked. You may give her that, but nothing else till I see her again." Next day the doctor looked in, and was surprised at the gloomy looks of the generally cheerful mother. "It's nearly all over with Kate, I'm thinking, doctor; she's been that sick and bad all night I thought she'd 'ave died." "Why, how's that? Mrs. Jones. I am sure she was doing famously yesterday." "I'm feared as that cake you ordered wasn't just the best thing for a delicate girl like her." "Cake!" exclaimed the puzzled doctor, "Cake; why that's the last thing in the world I should give her." With some temper the mother rejoined, "You may say what you like, sir, but you know very well that you told me to give her nothing but sweet bread until you called again, and so I told the squire's lady. She seemed a bit surprised, but she gave me a beautiful rich cake, and Kate's eat every bit of it."

### MISTAKEN IDENTITY.

A photographed group of nurses frequently has a place in the press reports of a hospital function, and the recent opening of the new buildings at St. Thomas' Hospital was illustrated in *Black and White* by a well-posed party of neatly garbed figures. No doubt the wardmaids therein represented are most valuable servants, but that is no reason why they should be labelled as "a portrait group of the nurses." Neither their caps nor their carriage resemble in the slightest degree those of the Nightingale probationers and staff nurses at St. Thomas' Hospital, and if the ward servants are gratified by the reporter's inadvertence, the nursing staff can hardly be expected to exhibit unqualified satisfaction in this mistaken identity.

### GOOD WORK, DONE WELL.

Last week a largely-attended meeting of the ladies interested in the Ladies' Theatrical Guild was held on the stage of the Lyceum Theatre. The rapid progress made by the Guild, which now numbers 464 members, is as remarkable as the unostentatious way in which it works. A weekly Sewing Bee is held in London every week, and there are provincial Sewing Bees too, where much excellent work is turned out. Forty-one maternity cases have been assisted, and there are twenty-eight now on the books. A special feature of this kindly charity is its delicate reticence regarding the names of those who are assisted. Of course some excellent speeches were made at the meeting, and Miss Kate Carton remarked that the President, Miss Fanny Brough, "never undertakes more than she feels she can do, but she nearly always does more than she undertakes," a reputation which many women workers would be proud to achieve. The thanks of the meeting were given to the women journalists, amongst others, who had helped to make the Guild so successful.

### AT THE ALBERT HALL.

One of the most attractive scenes in the wonderful programme carried out at the Royal Albert Hall on the 23rd inst. by Dr. Barnardo's children, was that in which the babies took part—a group of little creatures, who came in, led or carried by their nurses, and who, at a signal, made for their own iron cots, and, scrambling up, climbed the rails triumphantly taking possession of the pretty nests. From under each pillow was drawn forth a nightgown, and the little creatures began to undress themselves most methodically. The nurses—who, by the way, were models of dainty neatness—helped to remove pinafores, frocks, and petticoats, leaving only neat little flannel undergarments, which were rapidly covered by the nightgowns. It was noteworthy that, although the clothes were all admirably simple in design, they fitted excellently, serving unintentionally as an object lesson on "Rational Dress of Babyhood."

### "A FAD OF THE LADY GUARDIANS."

"She was deprived of rest from Monday to Thursday, and for three weeks she had been kept almost a prisoner to the ward." This is the official report of the trained nurse's condition in the women's ward at the Cheltenham Workhouse, as given in the local press. The presence of thirty-eight bed-ridden patients, besides "emergency" cases, such as confinements and typhoids, explains the immediate need for securing another nurse. How do these poor helpless old women usually fare in the night? Urgent claims of certain patients, as we read, kept the day nurse without rest from the Monday to Thursday; such incessant work as would be a terrible strain on the powers of any woman, and how much more so on the responsible nurse whose "daily round and common task" embraced attendance on forty-six other patients. In the face of these hard facts nine guardians voted against the proposal to engage another nurse, one guardian going so far as to say that two nurses would not have enough to do, "they would be squabbling and falling out." The resignation of the present nurse, which was worded, "Owing to the strain laid upon me the duties cannot be carried out in an efficient manner," may be withdrawn if she has assurance of a qualified assistant. Nurse Lambert has evidently done her best for the patients, despite the grievous strain laid upon her. None of our readers will be inclined to agree with Mr. Margrett that "she had forfeited the confidence of the Board," nor with the comments on "the fads of lady guardians," regarding adequate nursing. When the assistance of women guardians is claimed by every board we shall hear of fewer sick paupers neglected and of far less overworking of patient infirmary nurses.

### AN EXCESS OF EXAMINATIONS.

"Now if you were ill yourself," was asked of the experienced head of a nurse training school, "what class of woman would you prefer to nurse you?" "An intelligent upper servant, certainly," was the unhesitating response. In a very short time it is to be feared, however, that this most useful description of nurse may cease to exist. All such may be forced out of the nursing ranks, even if they are ever permitted to enter them, by the ever-growing craze for examinations. When applied to practical work and every detail of the personal care of the sick, then examinations are admirable indeed. But just now "theory" threatens to crush out practice. Examinations for nurses are rapidly attaining equal importance with those in the medical department, with the certain result that the ordinary intelligent and womanly probationer has to give place to the student to whom nursing is merely another science to study, and not an art to be loved. The much-jeered at "smoothing of pillows," as well as the humoring of restless patients and the encouragement of weak-hearted ones may seem beneath the notice of the hard headed young women whose whole thoughts are centred on gaining a first-class diploma. In fact, working for the latter, necessitates close application to books, and leaves little opportunity for that loving study of humanity for which a nurse's life offers unusual scope. Nurses to succeed and prosper must, however, be taught to nurse patients, not to treat cases.

### THE RIGHTS OF THE POOR.

The Newton Abbott guardians, in admitting that the children in their workhouse require to be better looked after, have been satisfied to appoint an inmate as nurse to those under five, at a salary of £12 per annum. A guardian at the recent meeting ventured to ask why this woman was in the workhouse. But he only elicited the reply (according to the local press), that she was a not particularly bright young widow. However, the Board saw her, and considered her strong and intelligent, and appointed her. The question of an increase of the nursing staff was again warmly advocated by Dr. Ley, and few will gainsay the truth of his assertion that "when the sick poor came there they had a perfect right to proper nursing." As the matter of the nurses at Newton Abbott has been brought to the notice of the House of Commons there is every chance of its now receiving due consideration.

### WHAT IS "THE PLAGUE"?

This very natural question is asked by many nurses just now. The casual remarks on the visit of the Great Plague to London recorded in school histories of England seem to leave but vague impressions on most adult minds. It was apparently so greatly dreaded that its victims were frequently inhumanly shunned by their fellows! In fact, tales of the period shed a halo of virtue over anyone who did not run away from their stricken relatives or friends. To-day, on the contrary, telegrams from Canton state that "the conduct of the soldiers and of the municipal police in entering the houses, assisting to remove the dead, and thoroughly disinfecting the dwellings attacked, is beyond all praise." According to a correspondent of the *British Medical Journal*, "The symptoms of the Hong Kong epidemic have been marked by high fever from the very onset, with vomiting; upon this followed buboes, chiefly in the armpits and the front of the thigh, the swelling being exquisitely tender, and suppurating later. The brain symptoms have been unusually severe, being commonly marked by convulsions and stupor, going on to coma and severe delirium. Many of the patients have died quite suddenly and instantaneously from heart failure."

### AT DETROIT.

A model modern institution is that at Detroit, Michigan, which bears the title of "Grace Hospital," and the training school attached to it is working most satisfactorily under the superintendence of Miss E. Hibbard. Besides seven wards for adults there are two for children, the latter being particularly charming and furnished with every possible comfort and appliance for the small patients. The building leaves little to be desired as regards structure or ventilation, and a room for convalescents is greatly appreciated. The patients' clothes—those which they wear on arrival—are warehoused in lockers in the basement, each one bearing a number which corresponds with a bed in a ward, bedside lockers are also provided. Specially admirable is the operating theatre, which is glazed and has large high windows. The instrument-room adjoins, and is as perfect in its way as the rest of the well-finished hospital; electric light as well as gas is laid on throughout the building.

### SHORT ITEMS.

The second annual report of the Penarth Branch of the Queen's Jubilee Institute is an excellent one, and the work of the nurse has increased so considerably that the securing of a second nurse is under discussion.—Nurse Amy Slater was presented with a fitted hypodermic emergency case and other useful gifts from her fellow nurses on her departure from the West London Hospital.—The Convalescent Homes' Fund is proving a most valuable auxiliary to the Newcastle "Cathedral Nurses."—The homes at Hexham and Shotley Bridge seem to be fully appreciated. Sheets, jam, fruit, and vegetables will be welcome gifts at both places.—Sister Josephine Broderick has been appointed nurse at the Skibbereen Union Hospital, she was the only applicant for the position; and is, happily, reported to be a trained nurse.—The testimonial to Nurse Hinton has reached about £100, and it will be shortly presented to her.

### On General Nursing.

By Rowland Humphreys, M.R.C.S., L.R.C.P.Lond.

### XVIII.—PERITONITIS.

Peritonitis is one of those diseases in which the name of a symptom is used to describe the disease. The term peritonitis of course means inflammation of the peritoneum, a membrane which lines the abdominal cavity. The peritoneum is in the shape of a bag; each of the abdominal organs is covered by peritoneum in such a manner that it bulges the side of the bag in. None of them are placed in the cavity of the bag of peritoneum. Thus any fluid which obtains entrance to the peritoneal sac can flow to any part of it, and, if of a septic nature, it may directly irritate all parts of the peritoneum instead of the inflammation or irritation merely spreading "by continuity of tissue," as in other parts of the body; the inflammation therefore may start in all parts of the peritoneum at almost the same time, being only subject (1) to the general laws of hydrostatics by which fluids tend to gravitate to the lowest level; (2) to the fact that the inflammation of the peritoneum tends to limit itself by adhesions between the surrounding structures; these make a little sac in which the pus, &c., is pent up; (3) to the peculiar powers of the peritoneum in respect to the absorption of fluid and foreign bodies in its cavity. This last property, although very satisfactory so far as the peritoneum is concerned, is very bad for the rest of the body, as the septic products absorbed cause a very severe form of blood-poisoning, which in its turn is the main factor in the symptoms which are collectively characteristic of peritonitis. Other characteristics of the peritoneum are its great surface, which is said to be as large as that of the skin, its limited resistance to septic organisms, its very favourable conditions for healing, and the different degrees of vulnerability of its various parts, that which covers the intestines being the most sensitive, that which covers the abdominal wall and the liver the least so. Therefore the parts most exposed in the ordinary course of affairs to injury are the ones which are the hardiest, whilst the parts which are, unfortunately, most exposed to irritation or injury in disease are those which are most sensitive. Thus, a typhoid ulcer will perforate the intestine and so injure and expose to injury the most sensitive part of a very delicate structure; on the other hand, an attack of peritonitis from cold or exposure is decidedly rare.

It has been pointed out that peritonitis may be set up either by germ irritation or by "chemical" irritation. That is, the germs, which set up peritonitis in most cases, may do so either by their own proper action or by the products of their own vitality. Thus it has been found that fluids in which some varieties of germs have been growing will set up peritonitis as readily after the germs have all been killed as while they were alive.

It has been found that the intestines contain throughout their whole course certain bacilli which have the power of causing peritonitis. The especial germ which has this power, and which is always present in the intestine, is that known as the bacterium coli commune. This little vegetable organism is quite harmless under normal conditions, when the intestine is in a healthy state, but an attack of diarrhœa or other slight ailment, and, of course, all the greater affections of the intestine, are quite sufficient to make this bacterium turn unkindly upon its host, and become one of his deadliest enemies. It then acquires the property of being able to pass through the intestinal walls, assisted, undoubtedly, by some change in the condition of those structures, and entering the abdominal cavity it sets up peritonitis.

Other forms of germs are also able to cause inflammation of this structure; such are those which are found in erysipelas, in pneumonia, and in phthisis. Cancer will also cause it. Thus peritonitis consists of several parts, all of which have to be taken into account in treating it. It consists of the remote and the immediate cause; of the actual inflammation of the peritoneum, and of the septic poisoning resulting from the action of the immediate cause, the bacterium; and of the remote cause, the condition which permitted the ingress of the germs to the peritoneal cavity, whether perforation, hernia, abscess, or enteritis.

The different forms of peritonitis are principally due to the nature of the germ causing the attack. These germs have certain features in common and certain dissimilarities. Some tend, as tubercular peritonitis, to a chronic condition with much thickening and many adhesions; another form of peritonitis is that due to pyogenic cocci tending to the formation of pus, as in puerperal peritonitis, when they are nearly always found.

After an attack of peritonitis the membrane giving its name to the complaint becomes more or less used to it, so that the next attack, if another should occur, will not be so severe; the germs causing it are not able to exercise so much effect as in the first attack; in other words, the peritoneum has acquired a certain degree of immunity from the effects of the germ. This immunity is very striking and is another of the marked properties of the peritoneum. The immunity protects the body generally, as well as the structures more immediately involved.

Peritonitis may be described according to the germs which cause it, or it may be divided into acute and chronic forms, or it may be divided up according to the remote causes. The best classification seems to be that which takes into account not only the germ, as the immediate cause, but the remote cause, whether it be cold, direct injury, as by perforation of the bowel, or some inflammatory condition which has spread from a neighbouring organ and has attacked the peritoneum. Treves in his admirable Lettsomian lectures (published in the *British Medical Journal*, February and March, 1894), describes the disease according to its immediate causes; another classification places it under the following heads: Traumatic, perforation, direct irritation, secondary to other diseases, idiopathic, and contagious. The disease differs so markedly in its forms according to the remote cause, and this is so profoundly affected by the particular germ which gains access to the peritoneal cavity, that both of these have to be taken into consideration in any description of the disease as a whole.

Perhaps the simplest method, for the purposes of this article, is to divide up the forms of the disease according to the treatment required for each, putting those which of necessity require operative treatment under one head, those which may require it under another, and those which usually do not require it under the third heading.

1. "Cases which do not usually require, or which are unsuitable for operative treatment."

Under this heading will come not only those milder cases of peritonitis due to cold, injury, &c., but also those cases where the peritonitis tends to remain confined to one part of the peritoneal cavity.

Cold as a cause of peritonitis has been denied, and it is probable that it only acts as the remote cause by producing some change in the intestinal canal which allows the bacteria in the intestine to pass through its walls. Other causes are those which result in perforation of the intestine or stomach, or other viscus, but in which, from the weakness of the patient or other cause the patient cannot usually be operated on. Thus in typhoid fever ulceration of the intestine is always present, and in some cases the ulcer extends through the wall, and the contents of the intestine flow out into the peritoneum. In some cases the intestine is not actually perforated, but the inflammation extends from the ulcer to the peritoneum, and so sets up inflammation. The inflammatory extension in this and in similar cases is the saving of the patient, for the neighbouring intestine or abdominal wall becomes adherent to the inflamed part, and the intestine, even if actually perforated, does not leak into the abdominal cavity. This often takes place in other cases of circumscribed inflammation, as in gastric ulcer. Absolute rest, both as regards food and movement, is therefore of prime necessity in such cases.

### English Nurses in Brazil.

(By a Correspondent.)

RIO DE JANEIRO.

III.—The Yellow Fever Epidemic.

After a quiet winter and spring, during which a few changes took place, the matron returning to England, and another coming out, three more nurses were added to the staff. At the end of February, 1894, yellow fever burst upon us in its most terrible form, which is called the "marine type" here, but we should naturally say "malignant." It begun amongst the shipping, and a young English doctor went out on the bay and visited the ships daily, sending the suspicious cases ashore at once. Poor fellow ! he was one of the earliest victims of the disease ; long hours and exhaustion left him no chance to fight against it. He came to us directly he felt sick, but had already developed a fatal symptom (suppression of urine), and although he had the best medical skill in the place he shortly succumbed. It was the more sad as he was shortly to be married to a young Brazilian lady. Captains, officers, and apprentices simply poured into the wards until our available beds were all filled. Then commenced six weeks of such work ! We were sometimes on duty for 33 hours at a stretch, without half an hour's rest—such terrible cases of "black vomiting" and intense suffering. I want to try and particularise the disease in order to give an idea to any intending to come out and nurse it.

The general symptoms are intense pains in the head, nape of neck, right down the spine, particularly severe in the lumbar region, pains in limbs as in other fevers, and a strained, bloodshot appearance in eyes ; sometimes there is a history of rigor, but not always. Almost all persons regular in their bowels generally, are troubled with constipation at the commencement of the attack of fever ; vomiting sometimes occurs at the beginning, not always. It is safer then being of a bilious type ; later on it is much more serious, as it is generally followed by the much-dreaded black vomit.

It is rather difficult to describe, as almost every case differs, one man will come in with the general symptoms described, and temperature 104 Fahr., to be ordered a warm bath, a purge, ice-bag to head, to be sponged every four hours, or oftener if required, nothing but iced seltzer or iced water to drink, benzoate of napthol powders every four hours, and Murray magnesia, with perhaps a little digitalis or arsenicalis, and he will get well in five or six days. That is a typical straightforward case, and the patient passes plenty of urine, has his bowels well open, &c. Another man will come in with the same symptoms, go on apparently well for two or three days, then the urine becomes scanty, full of albumen, and at last it stops entirely ; temperature falls to 98 Fahr. There is no pain nor sickness, yet nothing will make the kidneys act ; sometimes a patient lasts two or even three days in this condition, then death comes, heralded by a fearful uremic convulsion lasting only a few minutes, or sometimes even an hour, terrible whilst it lasts. Three people are often required to hold a man in the bed. The saddest part of this stage is their seldom losing consciousness. They know they are lost men, yet are so quiet and brave about it, just waiting for the dreaded end. It is most pitiful to see the change coming on just before the final struggle. The condition of quiet despair is succeeded by an anxious look which comes over the face ; later on a flush overspreads it, and the eyes are like glass in their brightness. At this stage the convulsion may occur at any minute. It is quite heartbreaking to see the imploring expression of the eyes, and the way the poor souls grip and hold on to a nurse's hand. Just before the convulsion came on one poor fellow said to me, "It's awfully hard lines, isn't it, Sister ?" Such splendid looking men as so many of them are, too.

Again, another man may come in with not a very high temperature, but looking extremely yellow, and states he feels very little pain and rather thinks it "a farce" to come in at all. In twelve hours, perhaps, there is total suppression of urine, he becomes quite delirious, and dies raving mad before he has been in twenty-four hours. Then a patient comes with a temperature of 104 deg. ; great tenderness over liver. He is put on the usual treatment, and painted over liver with iodine ; about the third day he gets hiccough, commences the black vomit, which he simply gulps up spasmodically, splashing and covering everything quite uncontrollably. At the same time passes the same thing under him. The odour is most poisonous, and just think how horrible it must be to the patient. This will last twenty-four hours, no urine passing, and at last death brings relief. The terrible exhaustion caused by the hiccough and vomiting is most painful to witness, in addition to the other horrible sufferings.

There is one complication which was probably mistaken for suppression in the past, and that is paralysis of the bladder. The catheter is invaluable then, and in many real cases of suppression, hopes that it was only retention have been indulged in.

Another phase is hæmorrhage from the tongue, gums, and nose. This is a trying condition, it being so difficult to keep the mouth clean. We had two boys who both became comatose for twenty-four hours, then quite delirious ; both had black vomit and stools, yet both recovered.

To show what a treacherous disease yellow fever is, I will just note the case of a German captain, a splendid man to look at, who had a mild attack, and got on very nicely apparently. He was allowed to go to the bath-room for his warm bath, as his temperature had been normal for three days. On the third day, after he had been doing this, in the evening, without any apparent cause, his temperature rose to 104 ; at twelve p.m. it was 105. He said he had no pain, and at half-past one a slight noise was heard in his room, and we found he had got on to the stool, but was speechless, and perfectly helpless. We got him into bed and found he had passed about a pint of pure pus. The doctor was immediately summoned, and pronounced it excess of penicioca (pernicious fever). We took temperature at two a.m. and found it 107 Fahr., and the patient died at half-past two a.m. Often apparently light attacks end thus disastrously, so nurses can never feel any security about a single case in their hands.

One lady, who had had a rather severe attack, recovered sufficiently to sit in a chair, then developed intermittent fever, and had fearful "night sweats," which drenched everything. She, however, appeared likely to do well, when she suddenly had a severe rigor, her temperatue rose to 105 deg., peritonitis set in, and she died within twenty-four hours.

Now I think I have given enough illustrations of the various cases of this dreaded disease to show how uncertain is its course. These are all cases I have had in my wards, and, of course, there have been many more.

We found hot sponging very successful in many cases where cold failed.

When I tell you yellow fever nursing embraces cupping, sponging, baths, painting, blistering, mustard poultices to hands, feet, abdomen, and neck ; injections, ice bags, bleeding, and perpetual "drinks." When you think of all this, and add the medicines, powders, and so on that have to be given, you will understand that the nursing of yellow fever is no light pastime. The opinion here is unanimous that we did wonders in the hospital in saving so many lives. One of the shipping agents told me we sent out cured nine of his captains alone whom he had never expected to see again. Perhaps these things ought not to be mentioned, but it shows what

English nurses can do, and we are deeply thankful to think we could be of such service.

Of course there was a sequel to all this work. Just as the great rush was over the matron went down with yellow fever, and I also fell a victim; another sister had it, then one was ill with erysipelas. Fortunately we all recovered.

Such is our experience of "Yellow Jack." I hope these notes will be of use to some other sisters who may have to nurse it. Nothing that ever was said or written about it is more terrible than the reality. No wonder there is such a wholesale dread of it. It is most painful, depressing, and disappointing nursing, but someone must do it, so I have no wish to discourage others. We are told by old residents that this season has been the worst since 1887.

Our efforts are most gratefully appreciated by the captains and others whom we nursed. When we were lying sick ourselves several of our former patients came up to hear about the "sisters." One captain insisted upon seeing us, and really it was very touching to see this great giant with tears in his eyes and voice. When he crept into our room he was quite overcome, and in a most reverent manner he lifted our hands and kissed them. We had nursed his wife and others from his ship. We hear that from some ships captain, wife, officers, and apprentices, whole ships' crews, officers, captains, &c., have been swept away. There are ships in the bay without a creature left. Out of 90 captains or so, 40 have lost their lives; as to able seamen, it is terrible to think of the numbers. The mortality stood at 100 to 110 per day of the populatfon of Rio, ships included. We are under a promise only to take officers and apprentices; able seamen are supposed to go to the native hospitals.

### St. Sebastian.

St. Sebastian is the large yellow fever hospital this side of the bay. There is another on the opposite side. We visited the former just at the beginning of the epidemic, with the vain hope of getting some useful information.

It is a very fine hospital, with accommodation for 300 patients, built on piles, bricked and cemented over. The two principal large wards had each 80 beds in; it looked curious. There is a low partition along the centre, then four rows of beds. This seems a great number, but the ward is so large there is no want of air. There are smaller wards, also private ones, and certainly it all looked scrupulously clean; but it seemed to us that there ought to be a good staff of nurses looking after the poor fellows. We saw in all about 150, in all stages of the disease. Some were vomiting pure blood, others the dreadful black vomit. It all seemed so fearful that so little could be done for them. We spoke to some Englishmen, and they spoke most nicely of the treatment they had received. The medical superintendent told us he intends introducing trained nurses as soon as they can afford it. At present they have male nurses, and of course, although good in their way, they leave much to be desired.

I am writing this during my own convalescence up the mountains, where the evenings are long, so I trust you will excuse all errors. But do not think I am morbid; I have simply stated things as they have been. The great rush is over, and perhaps we shall not see such a time for years—I sincerely hope not. We are not afraid for ourselves, it is others we fear for.

### Minor Appointments.

Oswestry Workhouse.—Miss Mary Ann Morris has been appointed Nurse at this workhouse. She was formerly Nurse at Camberwell Workhouse and at Knighton Union, and District Nurse at the Oswestry Cottage Hospital.

### Notes from Germany.

By a Correspondent.

Professor Dr. A. Neisser is justly proud of the new dermatological clinic in Breslau, of which he is the director. Until the year 1870 there were but three universities—Munich, Würzburg, and Berlin—with special provision for patients of this class. Heinrich Köbner was the first to begin the work in Breslau. He was succeeded by Oskar Simon, and in 1882 by Professor Dr. A. Neisser, who became director of the clinic, and with the help of his medical colleagues and assistants has succeeded in bringing this particular branch into notice. In 1892 a separate dermatological institute was opened, which stands unrivalled in Germany. Previous to this year patients were treated in All Saints' Hospital. The building stands at the corner of Max and Thiergarten Strasse. It was at first proposed to combine the clinic for internal diseases with the dermatological, and this would not have been without advantages; but finding that a separate clinic would cost only a trifle more, the first plan was relinquished with the full approval of both directors.

The new structure is handsome and imposing. The wards, which contain seventy-seven beds for adults and twelve for children, occupy the whole of the first story and the side wings of the ground floor. They are divided into three classes. The wards for the first and second classes are all on the first floor. The only distinction made between the treatment of the two is that first-class patients occupy separate private rooms at an inclusive charge of 6-8 M. For this they receive their daily board, medicine, baths, etc. Second-class patients are treated in small wards containing not more than four beds at a cost of 4·50 M. There are four first-class and four second-class rooms. A room reserved for especially severe cases is fitted up with a bath, and the patient is wheeled up to it in his bed, and by a simple contrivance is transferred to it, and thus spared the torture of being touched. The operating room is also on the first storey, and is fitted up with every necessity and convenience that modern science can suggest. All the beds run on castors, the patients being wheeled to and fro in all comfort.

The third-class patients are accommodated in four wards on the first storey and four in the side wings on the ground floor. The women's wards are in the northern wing, the men's in the southern. These patients are charged 1·50 M. per day, if able to pay the sum; foreigners pay a fee of 2 M. Attached to each ward are two small rooms, one in which patients are bandaged and examined, another for the charge-nurse, provided with a window overlooking the ward. There are also large, pleasant sitting-rooms for the patients not confined to bed, and spacious grounds surrounding the hospital for those able to take exercise. Each ward is furnished with a large dining table, easy chairs, sofas, &c., which give a very comfortable appearance. The staircases are of granite. The entire building has been erected and furnished at the moderate cost of 319,000 M.

The chair at Vienna University of the late Professor Billroth, the eminent surgeon, has been filled by one of his disciples and former assistants, Professor Gussenbauer, of Liège and Prague Universities. He is regarded as a bold and skilful operator, and a learned writer on modern surgical problems.

A gentleman volunteer is at present serving his country as an attendant in the garrison Hospital of Spandau. He is a West Prussian, and belongs to a sect so strongly opposed to war that the military authorities have allowed him to fulfil his military duties in a more peaceful way.

A petition from Leipzig has been forwarded to the Reichstag praying for the admission of women into the medical profession. It has again been refused. They are allowed to study, but cannot take their degree. Many, however, are not deterred by this decision, but graduate at a Swiss university, and already Leipzig can boast of several lady doctors, who are a source of constant vexation and jealousy to their male colleagues.

### For Reading to the Sick.

THE THORN IN THE FLESH.

**Motto.**

Know how sublime a thing it is to suffer and be strong.
—*Longfellow.*

**Verses**

The path of sorrow, and that path alone,
Leads to the land where sorrow is unknown.
—*Cowper.*

Is it indeed a loss or is it gain?
His life is pain, and he has nought besides;
Most miserable must he be indeed
If this be wholly evil as it seems;
But if this be the hardest ill of all
For mortal flesh and heart to bear in peace,
It is the one comes straightest from God's hand.
—*H. H. K.*

We did amiss when we did wish it gone
And over; sorrows humanize our race;
Tears are the showers that fertilize the world;
And memory of things precious keepeth warm
The heart that once did hold them.
—*J. Ingelow.*

Grief may be joy misunderstood.
—*E. B. Browning.*

In the cruel fire of sorrow
Cast thy heart, do not faint or wail!
Let thy hand be firm and steady,
Do not let thy spirit quail!
But wait till the trial is over
And take thy heart back again;
For as gold is tried by fire
So a heart must be tried by pain.
—*A. Proctor.*

**Reading.**

If there had been anything better and more profitable to man's salvation than suffering, surely Christ would have it by word and example. —*Thos. à Kempis.*

He that is afraid of pain, is afraid of something that will always be in the world. But this is a failure in reverence and respect. —*Marcus Aurelius.*

Are there "thorns in the flesh" in these days? Oh yes, many a one. We need not seek far. Go into any hospital or infirmary, and you will see cases in plenty over whom the sad word incurable has been spoken; not cases of dangerous illness, but chronic maladies that are not very severe, but are not likely to be any better in this world. Or we may see people living active lives, who have never a full measure of health, but have some "thorn in the flesh," thorns that, like St. Paul's, can never be drawn out, but remain until death. Is your case such an one as this? If it is, you have, no doubt, like St. Paul, prayed earnestly for deliverance and cure. And yet no cure comes. Ah! do not let yourself sink down in despair, for God is saying the same to you that he said to St. Paul, and wants you to learn the same lesson that he learnt, and that is to do God's work bravely, notwithstanding a weak body. To depend, not on yourself, but on the stronger and mightier than you, even on Him Who has said "Without Me ye can do nothing." To accept the trial joyfully, knowing that the patient endurance is, in itself, something done for Christ. To show the world that the religion of Jesus Christ is worth something. for it enables you to rise above your trial, to overcome it, and work in spite of it, to live so near Jesus as to be filled with His strength, and to say "When I am weak then am I strong." And though your trial will remain, you will forget it more and more, as your soul gathers ever more and more of the power of Christ. And at last your weakness will be "made perfect" in His strength. *C. M. Hallett.*

### Craig Colony for Epileptics.

The Legislature of New York State has passed and the Governor signed the Bill establishing a colony for epileptics in that State. The colony is named after the late Oscar Craig, President for some years of the State Board of Charities. The Bill provides for the purchase of a tract of 1,875 acres of beautiful land in the Tenesee Valley, near Mount Morris, in Livingston County. This tract is all in one piece, well watered by brooks, and consisting of fine fields, woodland and orchards, and already provided with picturesquely-grouped buildings to the number of thirty-five. It has been a colony of the Shakers for twenty or thirty years, and is, therefore, perfectly adapted to its new use.

The law requires that all of the buildings put up should be on the village plan. A board of five managers is provided for, and these have already been appointed. Governor Flower, in order to make the new charity as ideal as possible, decided to select a specialist on nervous and mental diseases as one of the managers, so as to insure the best scientific treatment of patients and to keep the resident medical men in touch with all the latest developments in the pathology and treatment of epilepsy. He also appointed a lady residing within a few miles of the colony as one of the managers, in order that the women and children and general housekeeping can be kept under constant surveillance. In addition a lawyer, a homœopathic physician, and an editor were added to the board. The managers serve without salary, and meet at the colony once or oftener monthly. Having these ends in view, the Governor appointed as the board of managers: Dr. Frederick Peterson, of New York; Mrs. C. F. Wadsworth, of Genesee; G. M. Shull, of Mount Morris; Dr. C. E. Jones, of Albany; and W. H. Cuddeback, of Buffalo.

An important provision in the Bill is that the managers may accept any bequests of persons interested in the welfare of epileptics, and it is believed that many charitable wealthy people will build cottages upon the splendid sites on the tract to bear their names and exist as lasting memorials to their desire to serve humanity in this wise.

A medical superintendent, steward, matron, pathologist, nurses, school teachers, teachers of various industries and arts, and so on, are to be appointed as needed; but the colony will not be ready probably to receive patients before the autumn of 1895.

It is thought that the colony will ultimately number 1,500 to 2,000 members. As soon as possible the 600 epileptics in the county almshouses will be taken in charge. Later private patients will be received at prices corresponding to the accommodation asked for. It is sure to become self-supporting in the course of time, and to grow into an industrial and agricultural village that will more than rival the similar and famous colony at Bielefeld, Germany, upon which this is, to a certain extent, modelled.

At their organisation in Albany, on May 3rd, the board of managers made Dr. Frederick Peterson, of New York, president, and George M. Shull, of Mount Morris, N. Y., Secretary of the board.

### Exeter City Asylum.

The following nurses and attendants passed the examination for the Nursing Certificate of the Medico-Psychological Association last month: William Thomas Hart, John William Radford, John Warner, Philip Glanville, Caroline Dunn, Marion Dunn, James Thorne, Osborne Burton, Ellen R. Falvey, Charles Frederick Luscombe, Lucy Symes, Nellie Lucas, Eda Symes, Elizabeth Elliott, Joseph Edward Luscombe. Dr. A. Law Wade, Medical Snperintendent of the Somerset and Bath Asylum, acted as assessor at the examination.

### The Mechanism of Movement.

### V.—THE SPINAL COLUMN.

ALMOST more important than the skull, if possible, is the spine—that marvellous chain of bones, each different from the others, though each is a perfect bony ring with spines outside. These bones extend from the head to the bottom of the back, and form three distinct curves—outwards above, inwards in the middle of the back, and again outwards at the lower end. These bones are collectively called the "vertebræ," and man, in common with other animals possessing a backbone, is called a "vertebrate" animal, whilst those without backbones, such as worms, slugs, &c., are named "invertebrate" animals. The uses of the spine are: (1) To support the head; (2) to give a support at the back to the whole length of the trunk, and to protect the organs contained therein; and (3) its most important function of all, is to protect the spinal cord, which is quite as important to us, though in a somewhat different way, as is the brain. For convenience in describing them, these vertebræ, or spinal bones, are given special names—not each separate bone, but the seven extending from the top are called "cervical," from their supporting the head, the next twelve are called "dorsal," and the five lowest "lumbar."

It is a curious fact that in every vertebrate animal, with the exception of three only, there are exactly the same number of cervical vertebræ, *i.e.*, seven. Thus the long neck of the giraffe and the extremely short neck of a pig or a whale has respectively only seven. The two topmost bones have distinctive names, *i.e.*, "Atlas" is the one on which the skull immediately rests, and is so called because it supports the head, its name recalling the old myth of the ancient Greeks and Romans of a certain man named Atlas, who presumed to wage war with certain heathen gods, and was condemned for ever afterwards to support on his shoulders the whole weight of the world. Hence the bone which has to hold up our head has not inaptly been called "Atlas," for after all, if not our skull, at least the contents of it, constitute our "world," since we derive from it all that is really life for us—our intellectual and moral powers, and our capacity to enjoy and appreciate what is beautiful and good and pure around us.

The next bone to "Atlas" is called the "Axis," and is very peculiar in shape to enable it to perform a special duty. It possesses a tooth-like projection or peg, which acts as a pivot, on which the head works or turns from side to side, and hence the term "Axis."

Sometimes thoughtless people try to lift a child up in play by the head, which is an extremely dangerous thing to do, for if the head were lifted only a very little way off this peg the spinal cord within would be seriously damaged, and instant death might be the result. The same thing happens in hanging. Then uppermost vertebræ are dislocated, as it is called, that is, separated from one another, and moved out of their proper place, and the spinal cord, instead of being in a smooth canal, is injured by the sharp edge of the bone, and hence follows instantaneous death. A thoughtless person lifting up a child by the head literally hangs it.

With regard to the special form of the spine as a whole, something must be said. It again is most admirably and wonderfully adapted to the use for which it is intended. In the first place take the curves. Were it not for these all grace and dignity of movement and attitude would be lost, and we should be like wooden dolls. If we wanted to move so as to look at something on one side, or behind us, we could not turn slightly, as now, but should have to turn our whole body with much labour and trouble, exactly as if we were stiff with rheumatism.

Again, the spinal column is in 24 distinct pieces, or, more correctly, in 19, for in adults the last five, which are distinct bones in children, are welded together into one solid bone called the coccyx, and this gives it its great mobility and elasticity. Between each separate bone is a little pad of solid gristle, which has exactly the same use as have indiarubber buffers in a train, which prevent the carriages from jarring and knocking violently against one another and making the people in them uncomfortable and shaken. Were the spine in one solid bone, extending down the back from the head, we should not be able to jump from any height without feeling pain, due to the jarring of the soft and highly nervous substance forming the brain, and, indeed, we might do ourselves very serious harm, causing concussion of the brain. Thanks, however, to the construction of the spine, we can jump from heights without harming ourselves; and, in fact, very few people at all realise how much of this comfort is due to this arrangement of the buffers in their spine.

Then the ribs with the breast bone in front and the spine behind make a sort of basket containing those important organs, the two lungs, the heart, the liver, kidneys, and stomach, and provide an excellent protection for them. There are twelve ribs on each side, seven of which are called "true," and five "false" or "floating." The "true" ribs are attached to the spinal bone behind and to the breast bone in front by means of cartilage, which allows of movement during breathing. The "false" ribs are attached to the seventh rib in front, and so act more freely than do the others. It is needless to say that it is a popular delusion that a man has one rib fewer than a woman. This is absolutely untrue, and anatomy places it beyond a doubt that men and women alike have twelve ribs on each side.

### A Case for Assistance.

WE have received the following appeal from two ladies of long nursing experience, either of whom will be glad to receive any contributions and to give further particulars. We have most carefully investigated the case, and have ascertained that the nurse in distress is thoroughly deserving and in much need of help: "A lady, trained at King's College, London, and for many years filling the post of matron in London and provincial hospitals, is now, through misfortune and ill-health, rendered almost destitute. It is through no fault of her own that these calamities have overtaken her, which makes her case the more distressing. We have known her more than twenty years, and greatly desire to help her, but have not the means to do so entirely ourselves. She is at present struggling to live by means of a small shop at Cardiff. A former patient, whose gratitude to her for past kindness is so great that she has devoted herself to her, is with her, as she is unable to get about herself, but she cannot assist at all in pecuniary matters. We have good reason for feeling assured that if she could be helped to stock her little shop and free herself from debt she could make an independent living, and for this purpose we venture to appeal to the sympathy of other matrons and nurses who have been more fortunate to assist us in giving her a helping hand. Any further particulars we shall be pleased to furnish, and also to receive any donations, however small, on her behalf, addressed to us at The Home for Aged Mariners, Egremont, Cheshire. Signed, FRANCES A. BELL, formerly Lady Superintendent of Mildmay Nursing Institution, and of Netherfield Hospital for Infectious Diseases, Liverpool, and for the last eleven years Lady Superintendent of these homes]; MARY FARROW, for sixteen years Matron of the Royal Albert Hospital, Devonport, and after that five years at East Suffolk Hospital, Ipswich."

### Wants and Workers.

*Royal Hospital for Incurables, Putney Heath.*—Votes are earnestly solicited on behalf of Annie Eliza Hicks, aged 50, who is a candidate for pension. Her health is too bad to permit of her doing anything for her own support, and a son, on whom she has principally depended for many years, has recently died of consumpton. The case is well recommended.

### Two Hours Off Duty.

AROUND GUY'S HOSPITAL.—I.

The outdoor recreation of a nurse in a metropolitan training school must necessarily be "of the streets streety," but it does not therefore follow that it need be prosaic or uninteresting. Indeed, immediately upon leaving the gates of Guy's Hospital, for example, she will find herself in the midst of scenes crowded by historical and other pleasant associations.

In the fourth year of King Richard II.'s reign, a man fell asleep, and dreamt that going into the fields to gather flowers he encountered innumerable monsters.

"Scattered crowds of the common people . . . struck with the curse of God, were changed into wild beasts. Asses, disdaining the curb, rose like wild lions to seek their prey, and leaping about the fields terrified all the citizens. . . . They would no longer carry sacks into the town, nor bend their backs to any burden; they claimed to be lodged and combed like horses. So the ass leapt higher than the leopard, and carried a longer tail than the lion himself. . . . The ox is become a lion; ox is a leopard; ox is a bear; but his old character of ox he hath forgotten. . . . Among the beasts was a boar, whom Kent produced, with shining eyes, breathing out flames, from whose burning no house is far enough removed for safety; lightning flies from his mouth; the blast of it a city burns, and he prepares for the fight with elephantine tusks."

Under this figure the poet Gower, in his dog-Latin "Vox Clamautis," prefaces his description of Wat Tyler's rebellion, when the mob, on the Feast of Corpus Christi, surging from the neighbourhood where at present Guy's Hospital stands, towards the Tower across the river, cut the throat of Simon Sudbury, the Primate of All England, and spread murder and rapine through the whole capital.

Gower, unlike most poets, seems to have been a prosperous man. When he was nearly seventy, on January 25th, 1397, he was married to Agnes Groundolf "by the chaplain of their parish church of St. Mary Magdalene, Southwark; not in the parish church itself, but in a chapel of Gower's own, under his quarters in the priory of St. Mary Overies."

St. Mary-over-the-River was originally a priory of nuns, founded by a "Mary" who kept a ferry-boat there before London Bridge was built. She endowed the convent with proceeds of the ferry. To build a bridge was always accounted a "work of charity" in the dark ages; bishops granted indulgences to those who constructed or maintained a bridge, more especially when it happened to be on the route of a pilgrimage.

The nuns of St. Mary Overies gave place to the Canons Regular in 1106. A fire in 1207 destroyed the priory, and when it was rebuilt in the reigns of Richard II. and Henry IV., Gower, the poet, was a most liberal contributor to the expense. He had a lodging of his own and a private chapel in the new monastery, dwelling here as a "borel clerk" until in his old age he was smitten with the charms of Dame Agnes Groundolf.

After his marriage he became blind. He seems to have continued to live quietly in the monastic lodging with his wife, and at his death was surrounded by the clergy, who honoured him as a great benefactor. His will is a good example of mediæval piety and charity. He bequeathed his soul to God and his body to be buried in St. Mary Overies.

He left 13s. 4d. to each of the four parish churches of Southwark for ornaments and lights, and 6s. 8d. to the curates for their prayers.

He directed that 40s. should be paid to the Master of St. Thomas's Hospital, 6s. 8d. to each priest attached to it, 3s. 4d. to each sister, 20 pence to every nurse, and 12 pence to each sick person under the care of the hospital, that one and all might give his departed soul the advantage of their prayers. He remembered St. Mary's Hospital, Westminster, also, and bequeathed 10s. to each leper house in London.

St. Mary Overies was bought from Henry VIII. by the people of Southwark for a parish church. It is now known as St. Saviour's, and not the least interesting of its many relics of the past is the fine monument to John Gower. The poet, in effigy, lies with his head resting on three volumes, inscribed with the names of his works: "Speculum Meditantes," "Vox Clamautis," and "Confissio Clemautis"; he is represented as wearing a collar from which hangs the device of Henry of Lancaster—the swan—which he probably received from the king as an expression of the royal pleasure in his poems. Mercy, Pity, and Charity are painted in the canopy with three appropriate devices: "Eu toy qui est filz de Dieu le pere Sauve soit que gist souz cest piere," "O bon Jesu fait la mercie a l'alme dont le corpse gist icy," "Pour ta pité Jesu regarde et met cest alme en sauve garde."

Offerings for the rebuilding of the priory in 1275, and of the chapel by Peter de Rupebus somewhat earlier, must have poured in from far and wide, so at least one would gather from the fact that the Archbishop of York granted eighty days' indulgence to all participators in the good work.

That curiously interesting man who set his mark so indelibly on his time, Cardinal Beaufort, is connected with Southwark; he had a palace here in his capacity of Bishop of Winchester, his arms may still be seen on a pillar in St. Saviour's Church. Johanna Beaufort, another member of Catherine Lynford's family, was married on Candlemas Day, 1424-5, in this church to King James I. of Scotland—a picturesque ceremonial his nuptial mass must have been, and the marriage feast was afterwards held at the episcopal palace hard by.

### Sentence Passed on "Nurse" Gillespie.

On June 20th Ellen Gillespie was sentenced at Chelmsford to five years' penal servitude for her cruelty to the unhappy children placed under her care in the Hackney Training Schools at Brentwood. The Judge (Mr. Justice Day), before pronouncing sentence, remarked, in the course of an admirable speech, "It is fortunate that such iniquities should be brought to light . . . it may tend to induce . . . those in authority over public institutions to exercise some little care, some little conscientiousness, some little diligence in the discharge of extremely important duties—duties which they so lightly assume and so slothfully discharge in too many cases. . . I do hope and trust in the interest of the public that the Government may institute a most thorough and searching inquiry into the whole of this melancholy business, because I feel that I have only to deal with one person, and that person, although primarily guilty, is not the only person who should be made to suffer in some way."

### Novelties for Nurses.

Patterns of delightful pure woollen materials have reached us from Messrs. Roberts, Harrison, and Co., Castle Donnington, and the accompanying catalogue is a most instructive one. We frankly own that we had little idea before of the wonderful variety of underwear from which suitable garments can be selected, at a variety of prices, for men and women, boys and girls. Nurses and many others will be glad to learn that they can obtain ribbed ends to add to their shrunken vest sleeves for a few pence, as well as night-dresses, dressing gowns, bodices, &c. We should advise our readers to send for a catalogue and see the attractive price list for themselves.

☞ For The Muses' Looking Glass, Everybody's Opinion, Book World for Women and Nurses, &c., see page cxxix. et seq.

### The Muses' Looking Glass.

### THE TOWER BRIDGE.

On Saturday the opening of the Tower Bridge takes place—a state pageant the like of which has not been witnessed for many years. The gloom which haunts the very name of the Tower will be for a day dispelled, and the old stern fortress will put on its holiday attire, and in the gala scene spectators will find but little reminder of past dark days of historic tradition. As to the bridge itself, of which we hear so much and understand so little, it will be worth our while, perhaps, to glance at the outline of its history and its construction—the latter in outline only, for the magnitude of the works requires, if stated in full, a longer notice than our present space affords.

Our information on this subject comes direct to us from headquarters, on the authority of Mr. Tuit, chief engineer to Sir William Arrol and Co., contractors for one section of this colossal undertaking. It was only quite lately Mr. Tuit brought out a book* where he describes much that is of real interest to us all just now about the bridge. Its actual technique it is unlikely any but a practical engineer can understand, but there is much which is to be understood and appreciated by us all in the publication. The frontispiece to the book is by C. W. Wyllie—the artist who above all others has caught the spirit of, and studied and loved our mighty river; not the river of leisure hours in its upper reaches, but the great ships' waterway, the crowded, mercantile, serious Thames which flows where little leisure is. Half hidden away as the river is from the pleasure - loving denizens of Belgravia and Mayfair, yet here is the secret of the vast metropolis, here lies its power, its strength. Not long ago a great French writer told us how the Thames, and the wharves, and the docks had all impressed his mind as nothing else in London had done; it had an interest as M. Zola most rightly insisted above any footway in the vast area of the City.

And from the river one passes to its bridges. Time and tide have done away with the earlier "Old London Bridge," the first permanent (?) bridge constructed over the Thames in these parts—one which has long ceased to be. An old stone arch bridge it was, typical alike of the twelfth century engineering, as of the simplified traffic of those days. And yet we are reminded that the triumph of a bridge at all in those times was, perhaps, a greater one, when engineers were only feeling their way, with no past experience to act as guide or warning, than is the latest and magnificent outcome of our accumulated knowledge.

Earlier than this, there is nothing to lead us to surmise any bridge was in existence until after the time of the Romans. Their ancient Watling Street, and what is known as Southwark, being connected in those days with a ferry, worked by oars. Mr. Tuit tells us that there is a suggestion of a bridge in the records of the attack on London in 994, by Sweyn, King of Denmark, but this is only recorded in a fact that many of Sweyn's men were drowned "because they took no notice of the bridge." In 993 another Scandinavian monarch, King Oulaf, of mighty memory, is chronicled to have sailed up the river as far as Staines without interruption. But between this and 1016 a bridge of some kind had to be built, as it is recorded that Etheldred, who died that year, fixed the tolls to be paid by all ships coming up to it.

In 1176 one of the clergy of London, chaplain of the church in the Poultry in which Thomas à Becket was baptised, proposed to build a stone bridge across the river, and we read that contributions towards its cost were made by the King and generous citizens, and even by the people throughout the whole country. This took thirty years to build, but so thoroughly was the work accomplished, that it remained intact for no less a period than seven hundred years. On one of the piers there was caused a chapel to be erected, dedicated to St. Thomas of Canterbury. In those times, as we may remember, it was the custom to build chapels on bridges; many examples of such remain to this day. The necessary funds for maintaining the bridge were derived from lands with which the structure was endowed, and certain monks were charged with the services in the chapel. The revenues derived from them have not only been sufficient, we learn on Mr. Tuit's authority, to enable the Corporation of London to rebuild the bridge, but also to construct another at Blackfriars, to acquire and free from toll Southwark Bridge, and, finally, to build the new bridge just completed at the Tower of London.

And now, having glanced at earlier history, we must turn our attention to this, the latest of the bridges on the Thames, the foundation-stone of which was laid on June 21st, 1886, by H.R.H. the Prince of Wales. The Tower Bridge has, therefore, taken eight years to build. It is, in appearance, unlike any structure that has yet appeared. The originality exhibited in the design is shown particularly, as the engineer remarks, in combining heavy steelwork with masonry of elaborate architectural character, the provision of high-level footways for use when the central portion of the bridge is open for the river traffic, and the application of the bascule principle on a scale never before attempted. The hydraulic pressure which is used for the opening, as for other purposes, is generated near the accumulator house, built on the east side of the southern approach, and was constructed by Sir William Armstrong, Mitchell, and Co., of Newcastle-on-Tyne. Every precaution has been taken so that the opening and shutting of the bridge shall be rendered as safe as possible, and so far, in all the tests it has been put through, the wonderful piece of engineering has worked admirably, and it would seem that no design would have answered the purpose better than the one selected. For many designs, it appears, were sent in to the committee before final selection—plans for bridges of every description and order, most of which Mr. Tuit describes in detail in his late work on the subject.

### Notes and Queries.

### Queries.

(88) *Strangers' Hospital.*—Can anyone give me the address of the lady in London who engages nurses for this hospital.—*A. C.*

(89) *Probationer.*—Please tell me whether a domestic servant would have any chance of being received into a hospital as probationer.—*Housemaid.*

(90) *India.*—Please give me some information about Lady Roberts' nurses.—*Enquirer.*

(91) *Up-country Nursing.*—Please inform me where I can apply for information.—*An American Graduate.*

(92) *Training.*—Where can I obtain one year's training in a London hospital?—*Glasgow.*

(93) *Holiday Homes.*—I should be grateful for information about Holiday Homes, where a nurse would not have too many rules to keep. —*Enquirer.*

### Answers.

(88) *Strangers' Hospital* (*A. C.*)—You can write direct to the Matron, at the Strangers' Hospital, Rio de Janeiro, Brazil, for particulars, or apply to the Matron of St. Bartholomew's Hospital, London.

(89) *Probationer* (*Housemaid*).—Good upper servants make admirable nurses, but it is necessary that they should be sufficiently educated to pass the examinations of the training school. If you write to the matron of a hospital or infirmary and ask for a copy of the rules, you will see what qualifications are required in a probationer.

(90) *India* (*Enquirer*).—See The Hospital of July 1st, 1893, page cxxxvi.

(91) *Up-country Nursing* (*An American Graduate*).—The hon. treasurer is the Hon. Mrs. Neville Lyttelton, 21, Carlton House Terrace, London, and the hon. secretary is Major-General Bonus, R.E., The Cedars, Strawberry Hill.

(92) *Training* (*Glasgow*).—See "How to Become a Nurse," by Honnor Morten, price 2s. 6d., 428, Strand.

(93) *Holiday Homes* (*Enquirer*).—There is a Convalescent Home at Bognor, two hours' rail from London, "For the wives, widows, or daughters of gentlemen." It is very comfortable. Particulars to be obtained from the Clerk at Merchant Taylors' Company, 30, Threadneedle Street, London.—At Brighton, either Crescent Houses, 90, Marine Parade, or Home of Rest for Nurses, at 12, Sussex Square, might suit you.

### Everybody's Opinion.

[Correspondence on all subjects is invited, but we cannot in any way be responsible for the opinions expressed by our correspondents. No communications can be entertained if the name and address of the correspondent is not given, or unless one side of the paper only be written on.]

### DISTRICT NURSES.

Miss Florence Saunders writes: I must protest against Miss M. Mason's views as to the qualifications necessary for a district nurse. After eight years' experience I feel more than ever convinced of the necessity of a thorough training for our nurses. Does Miss Mason really consider that a young woman with three months' experience is capable of nursing cases of pneumonia, pleurisy, acute rheumatism, or of managing bad confinements, when from careless or ignorant midwifery the child's sight or the mother's life are perhaps endangered? These are the cases which any district nursing association can show by its report are entrusted to its care. Though Miss Mason's "young woman" had ever so good a "head on her shoulders," she might, from sheer inexperience, lose it on an emergency. We want thoroughly trained women; and besides the hospital training, a district nurse should, if possible, have six months in one of the district training homes to test her capabilities and sense of responsibility. The best hospital nurses, the quickest at waiting on the surgeons (even the good ovarian nurse mentioned) will be a complete failure in the district unless she is the right woman for the post. A district nurse must have a thorough love of humanity for the foundation of her work; she must have tact, refinement, natural dignity, and an inexhaustible fund of sympathy for all the little ins and outs of the home life of the poor, with whom she comes daily in contact. Only those who have worked in these homes as nurses know of the multitude of little difficulties that have to be contended with. How necessary it is to be firm, and yet to gain the confidence of the rough fathers and husbands, or the ignorant but well-meaning neighbours, who, if nurse knows her work, will be conciliated and taught to look after the patient in her absence. Miss Mason speaks of district nurses having less responsibility because they always "work under the doctors." I maintain that they have more responsibility. In the ward there are the resident medical officer, matron, and Sister to appeal to; in the district, until the doctor arrives, nurse has to use her own judgment, and to be responsible in any emergency. She is, in fact, more in the position of a sister supervising the work of others; only instead of a capable probationer she has to deal with the roughest possible material, and unless she succeeds in impressing those at hand with the importance of attending to the little details of nursing, the patient will probably suffer during her absence. There is no doubt that the wives and mothers are the right people to do the nursing, if they are at all capable. In our town at least we have had many most encouraging instances of the success the nurses have had in teaching relations to attend to their sick themselves, and also of saving homes from being broken up by the parents being taken to the hospital or union infirmary. The poor people are always most grateful, and take a particular pride in the fact of nurse being a real lady, which they are quick to discern. I do not think letters like Miss Mason's are calculated to help us superintendents in keeping up the standard of nursing in the homes of the poor; but happily we are helped by the Jubilee Institute, and our very kind and able inspector, Miss Peters. We certainly want it to be more clearly understood than it is, that not every good hospital nurse can be a success in district work.

### THE GOLD CURE.

"A Nurse" writes: Can anyone kindly inform me whether there is any institution in or near London where inebriates are treated with the gold cure, and also if any satisfactory cases of cures have been authenticated?

### HAY FEVER.

"A Victim" writes: Can some reader of The Hospital tell me if persons subject to hay fever are less liable to it in India than in England? Any personal experiences on this matter would be gratefully received.

### MISSING ADDRESS.

A Nurse has applied to us requesting an immediate answer regarding a certain post at a London dispensary. Her letter, being unaccompanied by her name and address, cannot possibly be attended to.

### ADVICE WANTED.

"A Lady" writes: Can anyone inform me of an institution which would admit a girl of 15, dumb, helpless, and idiotic. She seems to hear what is said to her and to understand right from wrong. She is very clean in her habits. The father is a working man and has a hard struggle to keep a large family, and can ill afford to make any payment for this poor girl, who is the eldest of his family. I will gladly send further particulars to any Hospital reader who is able to help or to advise in this sad case.

### Where to Go.

A drawing-room meeting will be held on July 3rd in aid of the North-Eastern Hospital for Children at 2, Kensington Court, by Mrs. Athelstan Riley. The Bishop of Bedford will plead the cause of the hospital, Mrs. Arthur Stannard will read, and Miss Margaret Wild will perform two pianoforte solos.

A matinee in aid of the funds of the Royal Eye Hospital, Southwark, was held at the Lyceum Theatre last week. Seldom, if ever, has a greater attraction been offered by any programme in the furtherance of charity work. The authorities are to be congratulated not only on the excellence of the scheme, but on the success attending it. The theatre was crowded at three p.m., not even standing room to be had, and in spite of the drenching rain, the vestibule presented a bewildering scene of persons who had not previously secured their tickets clamouring for seats. In consequence of the fulness of the programme the matinee commenced at half-past two, when Miss Ellen Terry played Nance Oldfield's part in Charles Reade's comedy, a part in which the popular actress is more than usually attractive. This was followed by a recitation by Mr. Henry Irving, and a monologue by Mr. J. L. Toole. Mr. George Grossmith was especially appreciated in his humorous sketch of "The American Drama," and the songs he introduced into it. De Beriot's "Scène de Ballet" was rendered with much grace and finish by Miss Maud MacCarthy, a small performer on the violin aged about ten years. Mr. Tito Matteo's piano solo was much appreciated, as, indeed, were all the other items on the programme of this most brilliant entertainment. Funds, we remind our readers, are urgently needed for this much deserving charity, and in spite of the energy and zeal of the secretary and the officers of the institution, sufficient subscriptions and donations are not forthcoming. On the programme of the entertainment to which we have just been making reference this was especially brought home to all present, a picture of a ward closed for want of funds being represented on the cover, on which "A Mute Appeal" was inscribed; which, we heartily trust, may not appeal in vain.

### Queen's Nurse at Seacombe.

The Seacombe District Nursing Association was affiliated with the Queen's Jubilee Institute two years ago. It has a Queen's nurse of its own whose services are thoroughly appreciated by her poor patients. The Seacombe Association is not connected with any other institution, nor with the Cottage Hospital, as was erroneously asserted by a contribu[illegible]ecently.

### The Book World for Women and Nurses.

[We invite Correspondence, Criticism, Enquiries, and Notes on Books likely to interest Women and Nurses. Address, Editor, The Hospital (Nurses' Book World), 428, Strand, W.C.]

A Sketch of the Life and Character of Sarah Acland. (London: Seeley and Co. 1894.)

This is an interesting sketch of an interesting life, and those who knew the subject of the memoir and recall the influence for good which she exercised on certain sections of society in Oxford during the years she lived in that city, will derive much pleasure from the perusal of a biography which will remind them of days now, alas, long gone by. The number of those now living must be very small who were students at the time when Dr. Acland brought his bride to Oxford on his accepting the Readership in Anatomy attached to Christ Church, and who used to take part in the evening gatherings which met Sunday after Sunday in the Aclands' home. Many, however, will remember Mrs. Acland in later years, and while to those still resident in Oxford her memory is continuously preserved by the presence in their midst of the Sarah Acland Memorial Home, to others the issue of this little reminiscence will be very welcome.

The Gipsy Road. By Grenville A. J. Cole. (1 vol. Macmillan & Co., 1894.)

This little book will appeal to all who care for cycling. It is an account of a ride taken by Mr. Cole and a friend from Krakow to Coblentz through Hungary, Moravia and Bohemia. The work is illustrated by Mr. Edmund New with pen-and-ink drawings taken from sketches and photos by the author. Mr. Cole gives a very pleasant account of his ride and tells of many interesting things he noticed on his way. There is, of course, more of circumscribed detail than of general aspect in his descriptions, for touring on cycles necessitates keeping pretty closely to the roads. His remarks from time to time about the roads are not altogether encouraging to those who may feel disposed to follow in their wheel tracks, but on the whole they seem to have had a very good time. One of the most interesting chapters tells of the ride through Moravia, passing by Austerlitz, where the great battle of Austerlitz was fought, best known in detail to most of us through Count Tolstoï's great novel, "War and Peace." The unique scenery of the country is enthusiastically described, and the author speaks of the bright and picturesque dresses of the people. Mr. Cole seems to have found his tricycle rather unwieldy on some of the roads, and strongly recommends safety bicycles for rough touring.

The Jungle Book. By Rudyard Kipling (1 vol., McMillan and Co., 1894.)

The jungle book is a collection of charming imaginative tales of animal life in the jungle. Those who remember a story called "In the Rukh," one of Mr. Rudyard Kipling's "Many Inventions," will recognise in Mowgli the native ranger who was the foster-brother of wolves and knew all the secrets of the animals of the jungle. Mowgli was a small Indian child who was carried off into the jungle by Shere Khan, the lame tiger. Being chased, Shere Khan dropped Mowgli, who ran away, and was picked up by the big Father Wolf who took him to his cave. Here he made friends with the Mother Wolf and her cubs, and was brought up among them, and in due time was introduced to the rest of the jungle-folk and promoted to hunt with the pack. Shere Khan, robbed of his prey, plotted against Mowgli, the man-cub, and it called for all the protection of his friends to save him from the crafty tiger. Eventually Mowgli conquers Shere Khan by stratagem and sits and rejoices in the sun on the hide of his dead foe. Of the stories not telling of Mowgli, the most fascinating is that of "The White Seal," who goes in search of a home beyond the hunting reach of man. Mr. Rudyard Kipling shows immense knowledge of the habits of the animals about whom he tells these clever nonsense stories, and although professedly written for children "The Jungle Book" cannot fail to delight grown-up readers.

The Greater Glory. By Maarten Maartens.

It is with no small expectation of pleasure and interest that we take up a book by Maartens. We know, too, from experience the author's varied powers of introducing new scenes, and combinations of circumstances. Mr. Maartens tells us that his present story is one of high life—Dutch life, with the clinging savour of past times and traditions, bearing the stamp of truthful delineation. The history is occupied with the fortunes of two branches of the Reselaer family—rising and falling stars, so to speak. The author centres our sympathy on the family of the Baron, driven, through failing fortunes, to quit the beloved home of their race, and superseded almost by stratagem, and certainly by very ill-fate, by the upstart kinsman, Count Reselaer. All the characters, according to Maarten Maartens' wont, are strongly delineated and individual. The amiable Baron, of weak character and judgment; his wife, "the white Baroness," as she is called, with her gentle dignity and loving patronage of the poor; the unscrupulous kinsman, his exotic wife, and noble little son are a few of the prominent figures whose fates and fortunes we follow with deep interest to the close of the narrative. A great striving for power and position on the one hand, fraught with hard-earned success, but with bitter canker at the heart of him who attained the goal of glory; downfall and ruin on the other hand; but from the ruin new hopes and aspirations rise, phœnix-like, from the ashes. We close the volume, full of vivid life and incident, where the children of the two races take hands, and, leaving the lesser glory and strife of their surroundings, chose a path for themselves, such as they deem leads the way to greater glory.

Rural Hygiene is the title of a little pamphlet printed by Spottiswoode and Co., New Street Square (price 6d.) It consists of the very valuable address contributed by Miss Florence Nightingale to the Conference of Women Workers held at Leeds in November, 1893. We commend to our readers this excellent guide to health teaching in towns and villages.

### MAGAZINES.

The Girl's Own Paper.—The summer number of "The Girl's Own Paper" bears the seasonable title of "Mignonette," and representations of the flower form a pretty cover to the volume. This number contains the usual excellent stories, useful hints, nice illustrations, and varieties of all sorts.

### Books Received.

Charles Griffin and Co.
"Clinical Medicine." By Judson G. Burry, M.D.
"Year-Book of the Scientific Societies."

Digby, Long, and Co.
"In Due Season." By Agnes Goldwin.

H. K. Lewis.
"Analyses of Twelve Thousand Prescriptions." Compiled by W. Martindale, F.C.S.
"Gout, and Its Relations to Diseases of the Liver and Kidneys." By Robson Roose, M.D. Seventh edition.

Young J. Pentland.
"Handbook of Obstetric Nursing." By Francis Haultain, M.D., and James Haig Ferguson, M.D.

Kegan Paul, Trench, and Co.
"Manual of Hygiene." By I. White Wallis.

London and Universal Bank.
"Everybody's Pocket Cyclopædia." By Don Lemon.

**Periodicals and Pamphlets.**—The Westminster Review, The Vegetarian, The Harrogate Mineral Waters, The Beverley Recorder, The Charity Organization Review, Food and Sanitation, Midwives' Education and Registration, by William Carter; Uterine Drainage, by Moffat Flynn, F.R.C.S.; Guy's Hospital Gazette, Dry Methods of Sanitation, by G. W. Poore, M.D., Reich's Medicinal Anzeiger, The Strand Magazine, The Picture Magazine, Tit-Bits, Public Opinion, Engineering Record, Medical Record, Journal d'Hygiene, On Septic and Auto-Intoxications as Causes of Disease, by Dr. William Carter.

